# Targeting tumor-draining lymph node to overcome resistance to cancer immunotherapy: an update

**DOI:** 10.20517/cdr.2025.126

**Published:** 2025-10-24

**Authors:** Jianan Lu, Jiangnan Yu, Tuo Xu, Yina Li, Shuxian Chen, Qian Zhou, Lei Wang

**Affiliations:** International Cancer Center, Department of Immunology, Shenzhen University Medical School, Shenzhen 518054, Guangdong, China.; ^#^These authors contributed equally.

**Keywords:** Immune checkpoint inhibitor, immunotherapy resistance, tumor microenvironment, tumor-draining lymph node

## Abstract

Immune checkpoint inhibitor (ICI) resistance often stems from intratumoral T cell dysfunction. This review focuses on both tumor-intrinsic and tumor-draining lymph node (TDLN)-centric resistance mechanisms. We detail how specific defects within TDLNs - such as impaired dendritic cell migration and the establishment of immunosuppressive niches - initiate and perpetuate systemic immune dysfunction, ultimately leading to ICI resistance. To counter these challenges, we summarize the following TDLN-targeted strategies: (1) remodeling the TDLN immunosuppressive microenvironment to restore effective antigen presentation; (2) expanding the pool of progenitor exhausted T (Tpex) cells, with a focus on their primary reservoir in TDLNs; and (3) developing adoptive cell therapies using TDLN-derived Tpex cells to generate a robust, personalized antitumor response. By repositioning TDLNs as a central therapeutic target, recent findings suggest strategies aiming to overcome resistance at its source and improve ICI clinical outcomes.

## INTRODUCTION

In recent years, immune checkpoint inhibitors (ICIs) have achieved remarkable progress in the treatment of various malignancies by reversing T cell inhibition and restoring antitumor immune responses^[1]^. Particularly in melanoma, non-small cell lung cancer (NSCLC), and renal cell carcinoma (RCC), ICIs have demonstrated significant clinical efficacy^[2-4]^, representing a milestone in cancer therapy. However, the overall response rate remains below 30%^[5]^, and resistance, both primary and acquired, is frequently observed. Current studies have primarily focused on mechanisms within the tumor microenvironment (TME), such as excluded T cell infiltration, defective antigen presentation, interferon pathway disruption, and T cell exhaustion. While these findings have advanced our foundational understanding of ICI resistance, they largely overlook the “upstream regulatory mechanisms” governing T cell priming and differentiation.

Lymph nodes (LNs), as secondary lymphoid organs (SLOs), are critical hubs for T cell priming, fate determination, and long-term memory maintenance^[6-8]^, playing a central role in the early phases of the cancer-immunity cycle (CI Cycle). Emerging evidence suggests that tumor-associated LNs, particularly tumor-draining lymph nodes (TDLNs), are not only the initial sites for antigen presentation and T cell activation but also key battlegrounds for immune evasion^[9,10]^. Importantly, TDLN dysfunction, including impaired dendritic cell (DC) migration, vascular abnormalities, and accumulation of immunosuppressive cells, can lead to insufficient T cell priming, restricted effector T cell (Teff) egress, and T cell exhaustion, positioning TDLNs as critical sources of ICI resistance. Thus, reprogramming the LN microenvironment and enhancing the quality of T cell priming may offer a novel strategy to overcome ICI resistance at its source.

This review comprehensively summarizes the mechanisms by which T cell dysfunction contributes to ICI resistance, with a focus on the multifaceted role of LNs in regulating T cell immunity. Furthermore, we propose a TDLN-targeted immunomodulatory strategy and introduce a “TDLN-derived progenitor exhausted T (Tpex) cells” therapeutic concept based on tumor-infiltrating lymphocyte (TIL) therapy, offering a theoretical framework for advancing personalized precision immunotherapy aimed at reversing ICI resistance.

## RESISTANCE TO ICI

### Anti-programmed cell death protein 1/programmed death-ligand 1 monoclonal antibodies as ICI

**Figure 1:**
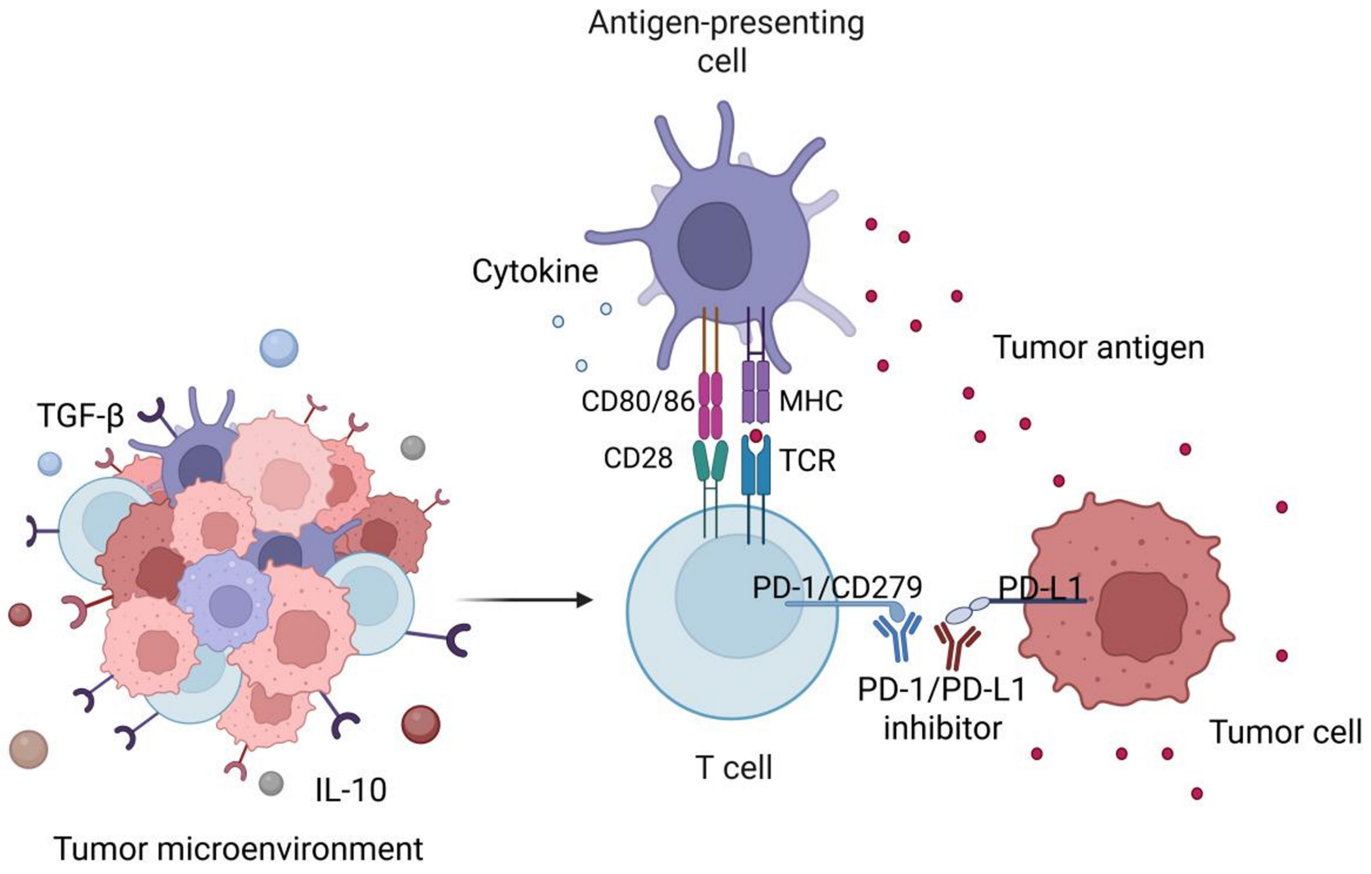
Mechanism of PD-1/PD-L1 inhibitors (ICIs) in the TME. APCs present tumor antigens to T cells through MHC-TCR interaction, accompanied by co-stimulatory signals (CD80/CD86) and cytokines that promote T cell activation. Within the TME, tumor cells overexpress PD-L1, which binds to PD-1/CD279 on activated T cells, suppressing their cytotoxic activity. PD-1/PD-L1 inhibitors block this inhibitory interaction, thereby restoring T cell activity, TIL function, and reactivating antitumor immune surveillance. Created in BioRender. Wang, L. (2025) https://BioRender.com/x410r92. PD-1: Programmed cell death protein-1; PD-L1: programmed death-ligand 1; ICIs: immune checkpoint inhibitors; TME: tumor microenvironment; APCs: antigen-presenting cells; MHC: major histocompatibility complex; TCR: T cell antigen receptor; TIL: tumor-infiltrating lymphocyte; TGF-β: transforming growth factor-β; IL-10: interleukin-10.

Programmed cell death protein 1 (PD-1/CD279) is an inhibitory receptor belonging to the CD28 family that modulates T cell activation^[11]^. It is primarily expressed on the surface of activated T cells and B cells, with a structure comprising an immunoglobulin-like extracellular domain, a transmembrane domain, and an intracellular signal transduction domain^[12]^. Its primary ligand, programmed death-ligand 1 (PD-L1) (CD274), is a type I transmembrane protein widely expressed on tumor cells and tumor-infiltrating immune cells^[13]^. In the TME, tumor cells bind to PD-1 via the high expression of PD-L1. This interaction recruits protein tyrosine phosphatase SHP-2, which can suppress T cell activation signals and facilitate immune evasion^[14]^. PD-1/PD-L1 inhibitors (e.g., pembrolizumab, nivolumab) block this binding, restoring the activity of cytotoxic T cells within tumors and enhancing TIL infiltration into the TME. Consequently, these inhibitors promote T cell-mediated antitumor immunity and reverse the immunosuppressive state^[15]^ [Figure 1].

### Primary and secondary resistance to ICI

Although ICI therapy has demonstrated significant efficacy in some cancer patients, its overall response rate remains low. Due to the complexity and heterogeneity of the TME, the benefits of ICI therapy are largely confined to specific cancers, such as melanoma, NSCLC, and RCC. Even within these indications, resistance remains a common clinical challenge. In 2020, the Society for Immunotherapy of Cancer (SITC) classified ICI resistance into primary and secondary resistance based on the timing of treatment response and provided explicit definitions, which will be expounded below^[16]^.

#### Primary resistance

Primary resistance refers to the absence of clinical benefit (e.g., no tumor shrinkage or disease stabilization) following PD-(L)1 inhibitor treatment. It typically manifests as disease progression during the initial treatment period (e.g., within 6 months). The underlying mechanisms include, but are not limited to, T cell activation impairment, tumor antigen presentation defects, and T cell infiltration impairment.

#### Secondary resistance

Secondary resistance refers to disease progression following an initial response to therapy (partial/complete remission or prolonged disease stabilization), emphasizing resistance that emerges after initial efficacy. This phenomenon may involve adaptive changes in the TME or genomic evolution. Secondary resistance arises through clonal selection or clonal evolution of tumor cells, where expanding clones acquire genetic alterations and have survival advantages that confer treatment resistance, also termed “acquired resistance”. Key mechanisms include mutations in the interferon-γ response genes [Janus kinase 1 (*JAK1*), Janus kinase 2 (*JAK2*)] leading to PD-L1 downregulation, as well as alterations in antigen presentation pathways (e.g., downregulation/loss of β2-microglobulin) resulting in defective antigen recognition. Other potential mechanisms of secondary resistance involve upregulation of alternative immune checkpoints [e.g., lymphocyte activation gene 3 (LAG-3) and T cell immunoglobulin and mucin domain 3 (TIM-3)]. Another general mechanism of secondary resistance also involves the expansion of immunosuppressive cell subsets [e.g., regulatory T cells (Tregs), myeloid-derived suppressor cells (MDSCs), M2-like macrophages] within the TME.

This review primarily explores the mechanistic framework of resistance to anti-PD-1/PD-L1 therapy, which involves primary resistance and secondary (adaptive) resistance. Notably, the emergence of drug resistance involves concerted actions of multiple mechanisms rather than a single factor. Therefore, we have only analyzed the complexity and diversity of resistance in general.

## RESISTANCE TO ICI CAUSED BY INTRATUMORAL T CELL DYSFUNCTION

Within the intricate defense system of tumor immunity, CD8^+^ cytotoxic T lymphocytes (CTLs) function as the primary effectors of the immune response. These specialized T cell subsets utilize their unique T cell receptors (TCRs) to precisely recognize tumor-associated antigens (TAAs) presented by major histocompatibility complex (MHC)-I molecules, a highly specific “lock and key” mechanism. Upon antigen engagement, CD8^+^ T cells initiate a dual cytotoxic program: (1) direct perforation of target cell membranes via perforin and granzyme release; and (2) FasL/Fas-mediated programmed cell death induction, thereby establishing a two-pronged attack against tumor cells.

Distinguished from the immune regulatory functions of CD4^+^ T helper cells, CD8^+^ T cells directly execute tumoricidal effector functions. Their activation process requires rigorous dual-signal validation (antigen signal and co-stimulatory signal) to ensure targeting precision. Emerging evidence indicates that the density of CD8^+^ T cell infiltration in the TME is significantly positively correlated with patient survival^[17,18]^, and their functional status is considered a pivotal biomarker for ICI efficacy. Notably, these cells possess immunological memory characteristics, enabling long-term tumor surveillance and playing a unique role in preventing tumor recurrence. It is this dual function of immediate killing and long-term monitoring that solidifies the central role of CD8^+^ T cells in cancer immunotherapy.

Although early studies primarily attributed ICI resistance to the absence of PD-1/PD-L1 expression within the TME, also known as “target absence resistance”^[19]^, clinical evidence has revealed a more complex and multi-layered picture. The antitumor response is a highly orchestrated process that encompasses T cell infiltration, tumor antigen recognition, activation-mediated killing, and eventual exhaustion. Dysfunction at any point within this cascade can precipitate immune evasion and confer resistance to therapy. Notably, these resistance mechanisms are highly context-dependent, exhibiting considerable heterogeneity across different cancer types. In RCC, for example, resistance often emerges despite robust CD8^+^ infiltration due to profound T cell exhaustion within the TME^[20]^. In contrast, lung squamous cell carcinoma predominantly employs a “spatial exclusion” strategy that prevents CD8^+^ T cell infiltration into the TME, thereby limiting ICI efficacy^[21]^. This review focuses on T cell-mediated tumoricidal mechanisms to elucidate potential origins of ICI resistance.

### Resistance to ICI by excluded T cell tumor infiltration

The efficacy of anti-PD-1 therapy is closely associated with the degree of tumor-infiltrating immune cells, where increased local T cell infiltration enhances antitumor responses. Multiple studies have confirmed that comprehensive TME classification models incorporating both PD-L1 expression and immune infiltration levels can effectively predict patient responses to ICI therapy^[22-25]^. These models categorize cancer into four distinct subtypes: Type I (PD-L1 low/TIL low), Type II (PD-L1 high/TIL high), Type III (PD-L1 low/TIL high), and Type IV (PD-L1 high/TIL low).

Type I tumors, commonly referred to as “cold tumors”, are characterized by low T cell infiltration, low tumor mutation burden (TMB), and minimal PD-L1 expression, along with abundant immunosuppressive populations such as tumor-associated macrophages (TAMs) and Tregs. These features collectively contribute to their poor responsiveness to ICI monotherapy^[26]^. Type II tumor is known as “hot tumors”^[23]^, characterized by robust T cell infiltration, high PD-L1 expression, elevated TMB, and abundant proinflammatory cytokines [including interferon (IFN)-γ, granzyme B (GrzB), tumor necrosis factor-α (TNF-α), and interleukin-2 (IL-2)]^[27]^, rendering them highly sensitive to anti-PD-1/PD-L1 therapy with optimal clinical outcomes. Type III tumors, despite harboring TILs, lack PD-1/PD-L1 expression. This phenotype is thought to reflect immune escape mechanisms mediated by adaptive resistance pathways independent of the PD-1/PD-L1 axis^[23]^. Within this context, TILs in the TME may fail to produce sufficient IFN-γ, often exhibiting features of T cell dysfunction or exhaustion. Type IV tumors, particularly prevalent in NSCLC, display high PD-L1 expression but minimal T cell infiltration. The absence of sufficient TILs undermines antitumor immune responses, resulting in poor efficacy of PD-1/PD-L1 monotherapy^[28]^. However, Type I, III, and IV tumors collectively account for over 50% of cancer cases and are typically poorly treated due to the lack of necessary targets for ICI treatment. This classification scheme provides a valuable framework for understanding tumor immune escape mechanisms and predicting immune therapy responses.

#### Chemokine recruitment dysregulation

The interaction between chemokines (e.g., CCL7, CXCL3, CXCL10, and CXCL6) on Teff cells and their receptors attracts effector CD8^+^ T cells to rapidly migrate to inflammatory sites or tumor tissues, amplifying the inflammatory response^[29]^. However, tumor cells can exploit CCR7 to suppress immune function and promote metastasis^[30]^. Studies have found that high expression of the CCR5/CCL5 axis in colorectal cancer (CRC) is significantly associated with increased infiltration of various immunosuppressive cells (e.g., Tregs, M2-like macrophages, MDSCs) and cancer-associated fibroblasts (CAFs), contributing to an immune-desert phenotype and potentially serving as a key mechanism of immunotherapy resistance^[31]^. Similarly, in KRAS-G12D point mutation models, the downregulation of chemokine CXCL10/CXCL11 secretion through reduced levels of high mobility group protein A2 (HMGA2) further disrupts immune cell positioning, leading to a decrease in CD8^+^ TIL^[32]^. Additional studies have identified the chemokine–receptor pairing of CXCR3 and its ligand CXCL9 as robust predictive biomarkers for responsiveness to anti-PD-1 therapy^[33]^. In a mouse model of metastatic melanoma, the combination of anti-CSFR1 antibodies and anti-PD-1 induced complete tumor regression, suggesting that T cell-derived colony-stimulating factor-1 (CSF1) enhances melanoma resistance to PD-1 blockade therapy^[34]^.

#### Intratumoral vascular structural abnormalities

Dysregulated expression of tumor suppressor molecules such as CD93 and its ligand IGFBP7 contributes to pathological tumor vasculature formation^[35]^. These abnormal vascular hinders leukocyte-endothelial cell interactions, creating a physical barrier that significantly impedes T cell infiltration into tumor sites^[36]^. Preclinical studies using murine CRC models have demonstrated that combinatorial therapy with anti-PD-1 agents and anti-angiogenic drugs can normalize tumor vasculature. This vascular remodeling modifies the TME to facilitate enhanced T cell trafficking and infiltration, ultimately potentiating antitumor immune responses^[37]^. At the same time, modulating macrophages in the TDLN to remodel vascular networks offers a means to promote Teff cell infiltration into tumors; further strategies will be elaborated in Chapter “Targeting macrophages in TDLN to remodel vascular networks”.

#### Carcinogenic signaling pathways constrain T cell infiltration

Activation of carcinogenic pathways represents a critical mechanism mediating T cell exclusion in tumors. The WNT/β-catenin pathway, for example, leads to the production of a large number of immunosuppressive cytokines, reducing the infiltration and function of CD8^+^ T cells^[38,39]^. This pathway additionally suppresses the recruitment of BATF3^+^ DCs and inhibits the secretion of the T cell-homing chemokines CXCL9/10^[40]^, thereby disrupting immune cell trafficking. Similarly, mutations in the PTEN tumor suppressor gene disrupt the phosphatidylinositol 3-kinase/protein kinase B/mechanistic target of rapamycin (PI3K/AKT/mTOR) signaling pathway, yielding a highly immunosuppressive microenvironment. PTEN deficiency promotes the expression of immunosuppressive cytokines and limits CD8^+^ T cell infiltration^[39]^. Notably, overactivation of cyclin-dependent kinase 4/6 (CDK4/6) promotes tumor cell proliferation and contributes to immune resistance, whereas the therapeutic use of CDK4/6 inhibitors (e.g., Palbociclib, Ribociclib, Abemaciclib) has been demonstrated to foster CD8^+^ T lymphocyte infiltration^[19]^, suggesting their potential in combination therapies.

#### Extracellular matrix fibrosis confines T cell penetration

Immunosuppressive cellular population, including MDSCs and M2-like TAMs, may inhibit T cell infiltration by secreting immunosuppressive molecules [e.g., transforming growth factor-β (TGF-β)^[41]^ and vascular endothelial-derived growth factor (VEGF)^[42]^], which collectively create an immunosuppressive microenvironment^[24,26]^. CAFs over-secreted collagen and depended on lysyl oxidase (LOX) cross-linking to form a dense network under the drive of TGF-β, PDGF, and other factors, leading to the remodeling of extracellular matrix (ECM)^[43]^. In addition, the abnormal activation of matrix metalloproteinases (MMPs) leads to the structural disruption of the ECM, which together hinders T cell penetration into the tumor parenchyma^[44]^. Ford *et al.* demonstrated in CAF-like phenotype tumor mice that NADPH oxidase 4 (NOX4) inhibition effectively reverses CAF-mediated CD8^+^ T cell exclusion, resulting in markedly improved responses to immunotherapy^[45]^. This finding highlights the therapeutic potential of targeting stromal components to overcome immune resistance.

#### Intratumoral metabolic stress suppresses T cell infiltration

**Figure 2:**
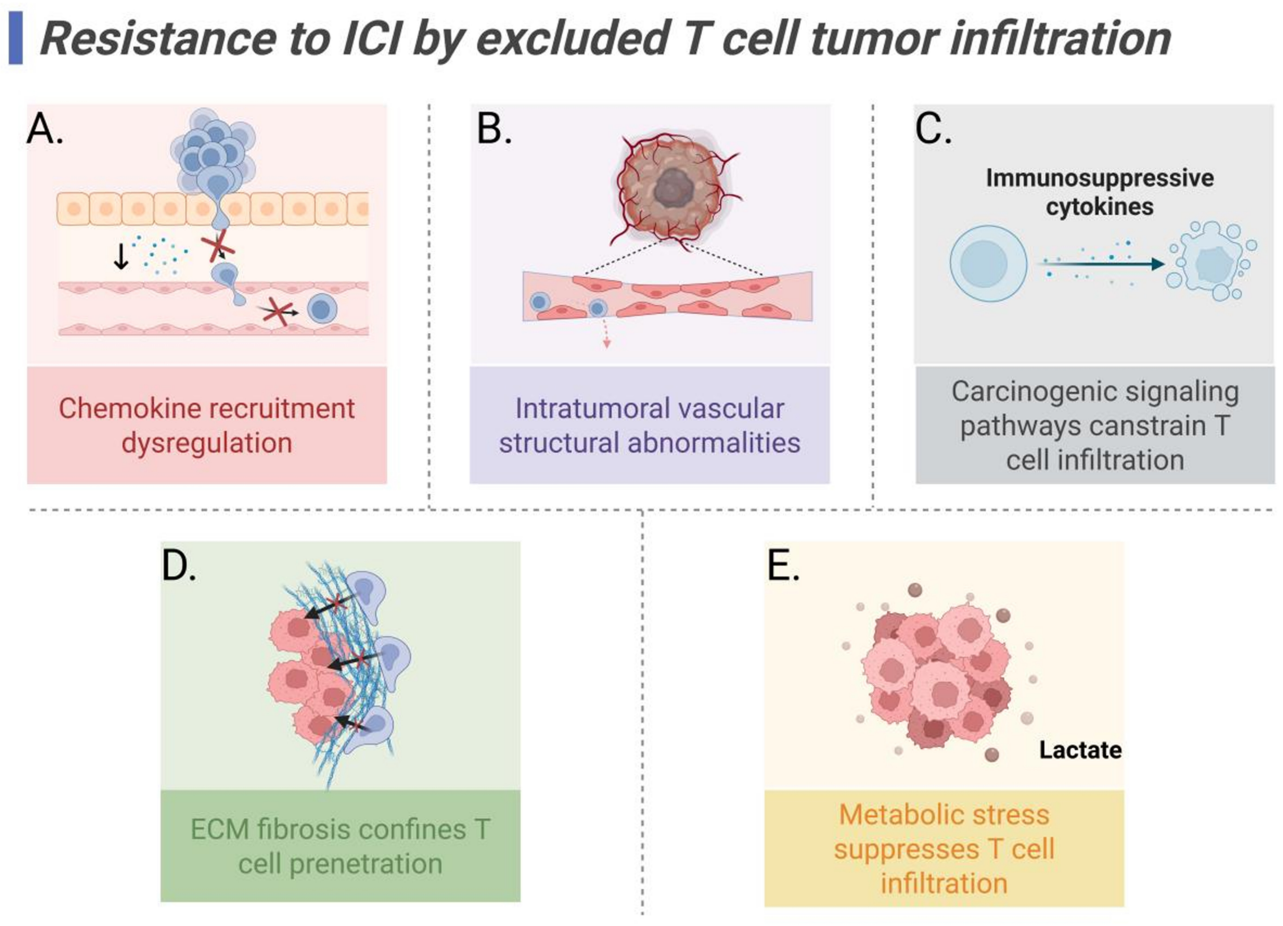
Mechanisms of ICI resistance mediated by excluded T cell tumor infiltration. (A) Chemokine axis dysregulation limits T cell recruitment; (B) Intratumoral vascular structural abnormalities hinder T cell trafficking and infiltration; (C) Immunosuppressive cytokine secretion driven by carcinogenic signaling pathways constrains T cell infiltration; (D) ECM fibrosis confines T cell penetration; (E) Intratumoral metabolic stress suppresses T cell infiltration, with lactate accumulation particularly impairing T cell metabolism and effector function, thereby limiting antitumor immunity. Created in BioRender. Wang, L. (2025) https://BioRender.com/5savrs6. ICI: Immune checkpoint inhibitor; ECM: extracellular matrix.

Metabolic reprogramming in the TME is increasingly recognized as a pivotal mediator of CD8^+^ T cell dysfunction and immunotherapy resistance. Highly glycolytic tumors consume vast amounts of glucose via upregulated glucose transporters (e.g., GLUT1) and glycolytic enzymes (e.g., LDH-A), leading to lactate accumulation and acidification of the microenvironment^[46]^. This induces metabolic stress on T cells and compromises the infiltration and activity of antitumor lymphocytes. In clinical studies of gastric cancer (GC), NSCLC, and melanoma^[47]^, elevated expression of glycolysis-related molecules lactate dehydrogenase A and oncogene c-Myc (MYC) correlated with resistance to PD-1 blockade. Currently, strategies targeting the lactate metabolism of tumors *in vivo* to restore immune suppression and promote immunotherapy are still under investigation^[48]^. Additional studies have shown that phospholipase A2 group X (PLA2G10) protein is overexpressed in immunogenic mouse tumors. PLA2G10 hydrolyzes phospholipids into small lipid metabolites, which suppress chemokine-mediated T cell migration and impair T cell infiltration, thereby conferring resistance to PD-1 immunotherapy^[49]^. Moreover, due to the high metabolic competition in TME, lactic acid accumulation leads to hypoxia in the microenvironment. Hypoxia enhances the expression of PD-L1 in tumor cells through the hypoxia-inducible factor 1-alpha (HIF-1α)/mTOR pathway, and activates G protein-coupled receptor (GPR81) through lactic acid, inhibiting the antigen-presenting ability of DCs and forming type I TME, namely “cold tumor” type^[50]^. High-fat diet-induced obesity alters fatty acid distribution in tumors, reducing the number of CD8^+^ T cells and impairing their infiltration and function. Metabolic reprogramming blockade in obese mouse models enhances antitumor immunity, suggesting metabolic intervention as a promising strategy to improve immunotherapy outcomes^[51]^ [Figure 2].

Beyond conventional CD8^+^ effector memory T cells, emerging evidence highlights the critical contributions of tissue-resident memory T cells (Trm) and peripheral T cell populations in replenishing TILs and sustaining antitumor responses during immunotherapy^[52-55]^. These findings further highlight the pivotal contribution of diverse T cell populations to the initiation and maintenance of antitumor immunity within the TME.

### Resistance to ICI by defective TAA recognition

T cell-mediated specific recognition of tumor cells constitutes the cornerstone of antitumor immune responses. It commences with the presentation of tumor antigens by MHC molecules on antigen-presenting cells (APCs) or tumor cell surfaces. CD8^+^ T cells recognize MHC-I antigenic peptide complexes through TCRs, whereas CD4^+^ T cells depend on MHC-II antigenic peptide interactions^[56]^. Effective recognition requires not only high-affinity TCR antigen interactions but also coordinated regulation by co-stimulatory molecules and cytokines. Local proinflammatory cytokines (e.g., IFN-γ) enhance antigen presentation by activating the Janus kinase/signal transducer and activator of transcription (JAK-STAT) signaling pathway, thereby upregulating MHC molecules and genes related to antigen processing [e.g., transporters associated with antigen processing (*TAP*), low molecular weight peptide (*LMP*)]^[57]^. Disruption of any component in this cascade impairs T cell “sensing” of tumor antigens, leading to immune surveillance failure and ICI therapy resistance. We can overcome defective recognition and presentation of TAAs either by targeting DCs in the TDLN to enhance T cell activation, or by employing personalized vaccines to strengthen T cell recognition of TAAs; detailed strategies will be presented in Chapters “Targeting DCs in TDLN to enhance antitumor T cell priming and activation” and “Targeting TDLN with personalized vaccines to enhance TAA recognition by T cells”.

This chapter outlines three primary causes of T cell recognition impairments, including the low immunogenicity of tumor antigens, epigenetic suppression of HLA-I molecules, and the absence of interferon signals. These factors collectively contribute to T cell inefficiency in recognizing tumor antigens, culminating in an “immune escape” phenotype and ultimately leading to resistance to ICI treatment.

#### Low immunogenicity of TAAs

The most fundamental cause of T cell recognition failure stems from insufficient tumor antigen availability, which directly compromises T cell-mediated tumor targeting^[58]^. Current research suggests that antigen heterogeneity and tumor genetic instability^[14]^ make it impossible for T cells to accurately identify and effectively kill tumor cells, resulting in the lack of efficacy of ICI therapy.

Through genetic or epigenetic changes, tumors can downregulate or lose the expression of immunogenic TAAs or tumor-specific neoantigens, leading to antigenic insufficiency and damaging the specificity of T cell recognition^[59,60]^. Clonal heterogeneous tumor cells may undergo immune selection^[24,38]^ during immunotherapy, leading to the loss or downregulation of previously expressed tumor antigens, thereby promoting acquired resistance to treatment^[60]^. The concept of TMB has emerged as an important parameter in this context, serving as a surrogate measure of a tumor’s capacity to generate neoantigens. High-TMB tumors tend to be more immunogenic, yielding more robust CD8^+^ T cell activity and stronger responses to ICI therapy^[29,61,62]^. In clinical trials across diverse malignancies, higher TMB reliably predicts better objective response rates (ORR) and improved clinical outcomes^[63-65]^.

Even when tumors express antigens that are distinct from those in normal tissue, low immunogenicity can still hamper an effective T cell-mediated response^[58,66]^. Conversely, tumors with high mutational load or microsatellite instability frequently produce highly immunogenic antigens that intensify T cell recognition and killing, yielding superior immunotherapeutic efficacy^[64,67]^. In low-immunogenic tumors, ICI responsiveness depends critically on the activity of CD8^+^ TCF1^+^ T cells, whereas in highly immunogenic tumors, therapeutic efficacy occurs independently of TCF1^[68]^. Preclinical studies have confirmed that deficient responses in low-immunogenic tumors can be rescued by personalized neoantigen vaccines, which restore antigen presentation and foster robust antitumor T cell activity^[69]^.

Moreover, chronic exposure to weak antigens may induce immune tolerance in the host, resulting in the production of exhausted T cells^[70]^ and further weakening the antitumor immune response. This phenomenon underscores the dual challenges of inadequate antigen priming and active immunosuppression in low-immunogenic TMEs.

#### Defective MHC-I antigen presentation

The ability of CD8^+^ T cells to recognize and kill tumor cells depends on the intact presentation of antigens by MHC-I molecules. The MHC-I molecules consist of a heavy chain (α chains) and a light chain (β2-microglobulin, β2M), with β2M being essential for proper MHC-I folding and surface expression. Multiple studies have shown that mutations in the *β2M* gene [point mutations, deletions, or loss of heterozygosity (LOH)]^[71,72]^ can lead to epigenetic silencing of MHC class I molecules^[73]^, preventing proper folding and transport to the cell surface, thereby inhibiting the presentation of tumor antigens to T cells. Sade-Feldman *et al.* identified that pre-existing β2M-LOH correlates with poor ICI response in melanoma and NSCLC^[71]^. Zaretsky *et al.* also found that melanoma patients who relapsed after 14.9 months of Pembrolizumab treatment had a 4-base deletion frameshift mutation in exon 1 of the *β2M* gene, which abolished MHC-I membrane localization, thus causing immune therapy resistance^[74]^. However, these findings are currently limited to melanoma and NSCLC, and the results have certain limitations, requiring further validation in other cancer types. Similarly, Fu *et al.* found that BIRC2 downregulates MHC-I expression by promoting ubiquitin-mediated degradation of NIK in hepatocellular carcinoma (HCC), and its inhibition in combination with ICIs enhances antitumor efficacy^[75]^.

The genotype of HLA molecules represents another critical factor influencing tumor antigen presentation defects. Studies have demonstrated that pre-existing β2M-LOH is associated with resistance to ICIs in melanoma and NSCLC^[72]^. Research by Chowell *et al.* further confirmed that HLA-I homozygosity is negatively correlated with ICI efficacy, which may be attributed to the fact that high HLA evolutionary divergence (HED) enhances the ability to recognize tumor antigen peptides^[76]^. The clinical significance of HED in predicting ICI response has been independently validated^[77]^, and the combination of HED with TMB can improve the predictive power of either feature.

In addition, the genes of HLA folding (*CANX* and *HSPA5*)^[78]^ and antigen processing and transport proteins (*TAP1*, *TAP2*, *TAPBP*, *CALR*, and *PDIA3*)^[79]^ also play an important role in antigen presentation and affect PD-1 treatment outcomes. Although these genetic alterations remain relatively uncommon in clinical settings, they further underscore the complex interplay between antigen processing and clinical outcomes.

#### Intratumoral dysregulated interferon signaling pathway

Interferons (IFNs) play pivotal roles in antitumor immunity by inducing antigen presentation and promoting CTL activity. IFN-γ, secreted by activated T cells, triggers the JAK/STAT pathway in tumor cells, upregulating PD-L1 expression and antigen-processing machinery^[80,81]^. However, chronic IFN-γ exposure can paradoxically dampen antitumor immunity, with sustained activation of the JAK/STAT pathway promoting resistance to ICIs^[82,83]^.

Genetic alterations in JAK1/2 or interferon receptors (IFNR1/IFNR2) render cells insensitive to interferon signaling^[24,38]^, thereby weakening the immune system’s recognition and response capabilities. Type I interferons (IFN-α/β) have been shown to induce NOS2 expression, which has been associated with the expansion of immunosuppressive Tregs and MDSCs, further blunting the antitumor response^[84]^. Additionally, the acidic environment of the TME in solid tumors causes pro inflammatory cytokine IFN-γ to denature and become inactivated^[85,86]^, constituting an additional factor in ICI treatment resistance.

**Figure 3:**
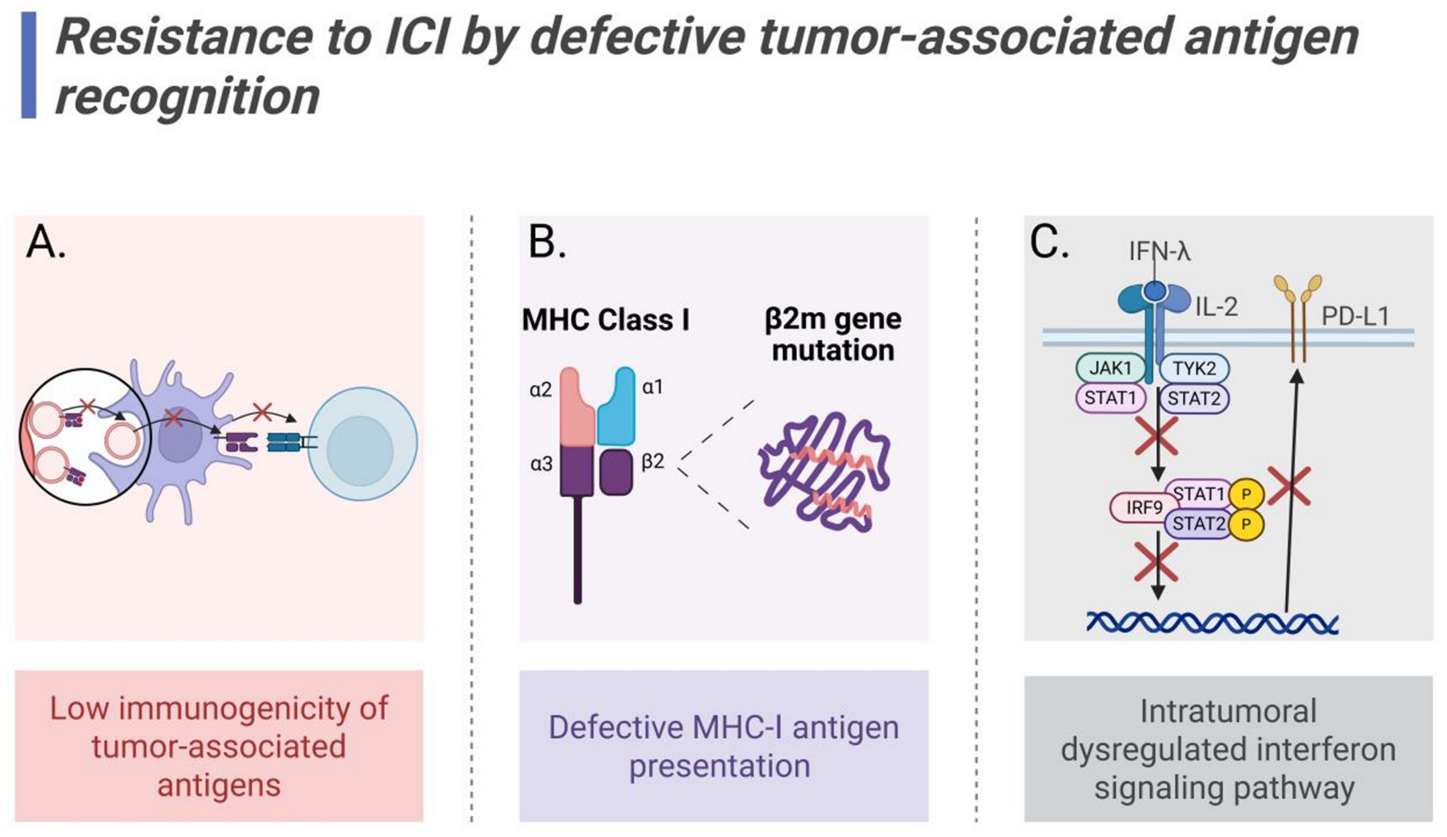
Mechanisms of ICI resistance mediated by defective antigen recognition. (A) Low immunogenicity of TAAs impairs T cell recognition; (B) β2m mutations induce antigen presentation defects; (C) Intratumoral dysregulated interferon signaling pathway downregulates PD-L1 expression. Created in BioRender. Wang, L. (2025) https://BioRender.com/j7gdp82. ICI: Immune checkpoint inhibitor; TAAs: tumor-associated antigens; PD-L1: programmed death-ligand 1; MHC: major histocompatibility complex; IFN: interferon-γ; IL-2: interleukin-2; JAK1: janus kinase 1; TYK2: tyrosine kinase 2; STAT1/2: signal transducer and activator of transcription 1/2; IRF9: interferon regulatory factor 9.

Hu *et al.* found in HCC that IFN-α remodels the TME through glycolytic reprogramming to activate immune responses, proposing combination therapy with PD-1 inhibitors as a potential strategy to overcome ICI resistance, though this approach remains exploratory and requires further clinical validation^[87]^. Interferon levels may serve as biomarkers for immunotherapy response across various cancer types^[88-90]^, with work by Boukhaled *et al.* demonstrating that pre-treatment resistance to IFN-I in peripheral blood CD4^+^ and CD8^+^ T cells positively correlates with long-term survival following PD-1 inhibitor therapy^[91]^ [Figure 3].

### Resistance to ICI mediated by intratumoral T cell exhaustion

Even if T cells successfully recognize antigens, their activation process may be subject to multiple inhibitions. Under chronic antigenic stimulation, persistent TCR signaling and downstream Ca^2+^ flux activate the transcription factor nuclear factor of activated T cells (NFAT), which in turn drives the expression of TOX and NR4A family transcription factors. NFAT-mediated signaling triggers the transcriptional upregulation of various inhibitory immune checkpoint proteins^[92]^, causing CD8^+^ T cells to gradually enter an exhausted state. The phenomenon of CD8^+^ Tex cells was first identified and characterized in a mouse infection model with lymphocytic choriomeningitis virus (LCMV)^[93]^. Research demonstrates that under chronic and sustained antigenic exposure (e.g., persistent infection or immunosuppressive TME) and metabolic stress, CD8^+^ T cells undergo functional impairment rather than apoptosis. These cells exhibit markedly reduced proliferative capacity and effector cytokine production, entering a reversible state of functional deficiency characterized by restricted effector potential and epigenetic reprogramming, ultimately failing to effectively eliminate antigens or tumor cells^[94]^.

#### Functional phenotypic features of exhausted T cells

The hallmark features of CD8^+^ Tex cells include persistent high expression of co-inhibitory receptors (e.g., PD-1, TIM-3, LAG-3, CTLA-4), diminished secretion of effector cytokines (e.g., IL-2, TNF-α, IFN-γ, GrzB) leading to impaired proliferative and cytotoxic functions, as well as metabolic reprogramming featuring mitochondrial dysfunction, glycolytic dependency, and aberrant amino acid metabolism. Concurrent epigenetic remodeling involves chromatin structural alterations, DNA methylation anomalies, and dysregulated histone modifications^[24,95]^. Notably, the co-expression pattern of multiple inhibitory receptors in CD8^+^ Tex cells reflects functional heterogeneity, where isolated expression of one or two inhibitory molecules does not fully define the exhausted phenotype^[96]^.

The process of T cell exhaustion ranges from Tpex cells (PD-1^+^, TCF1^+^, CXCR5^+^), which possess stem-like self-renewal capacity and multilineage differentiation potential, to terminally exhausted T cells (Tex-term; Tim3^+^, CD39^+^, TCF1^-^) that exhibit complete functional impairment and proliferative arrest^[97,98]^. Tpex cells demonstrate heightened sensitivity to ICI, with PD-1/PD-L1 inhibition promoting their expansion and differentiation into antitumor effector populations, making them primary responders to immunotherapy^[69,99]^. In contrast, Tex-term cells may contribute to immunosuppression. Clinical observations reveal that tumor-infiltrating Tpex proportions correlate positively with ICI efficacy, whereas Tex-term cells enrichment predicts therapeutic resistance. Thus, while CD8^+^ T cell exhaustion markers may indicate favorable treatment responses during early therapeutic phases, their persistence during late-stage treatment often portends poor outcomes^[100]^. Given the unique characteristics of Tpex, intratumoral Tex can be alleviated by expanding Tpex pools in the TDLN; detailed strategies will be discussed in Chapter “Expanding Tpex pools in TDLN to alleviate intratumoral T cell exhaustion”.

#### Overexpression of T cell co-inhibitory receptors

Advanced characterization of T cell exhaustion has revealed that proliferative impairment is closely associated with the upregulation of co-inhibitory receptors, which suppress TCR signaling and consequently diminish the durability of ICI responses. This represents a critical mechanism of resistance to cancer immunotherapy^[95,101,102]^. Studies in murine head and neck carcinoma models demonstrate that elevated TIM-3 expression in exhausted T cells, particularly Tex-term cells, attenuates T cell activation by inhibiting AKT/S6 phosphorylation, thereby compromising therapeutic efficacy^[103]^. Tex-term cells lose memory T cell characteristics, exhibiting a CD62L^-^ CD127^-^ phenotype. The ratio of effector memory to exhausted T cells within the TME may serve as a prognostic biomarker for patient survival and a predictor of immunotherapy response^[104]^. Exhausted T cells typically co-express multiple inhibitory receptors, whose signaling pathways may synergistically suppress T cell function. Monotherapy targeting a single inhibitory receptor (e.g., PD-1 blockade) fails to fully reverse exhaustion or restore T cell functionality. Clinical evidence shows that the combination of PD-1 and LAG-3 bispecific antibodies can improve antitumor responses; however, carefully evaluating the balance between efficacy and potential autoimmune risks remains crucial^[105]^.

#### Limited intratumoral proliferative IL-2 production

IL-2 is a key cytokine driving T cell expansion and effector function^[106]^. The decline of IL-2 is one of the earliest biomarkers in Tex cells, and therapeutic strategies targeting effector cytokines such as IL-2 may potentially reverse the exhausted phenotype. Multiple clinical trials investigating IL-2-based combination therapies with ICIs are currently underway, with phase I studies in solid tumors demonstrating promising clinical responses^[107]^.

A hallmark feature of Tex cells is the significant impairment of both effector function and proliferative capacity, ultimately compromising antitumor immunity. Notably, CD62L^+^ Tpex cells play a pivotal role in antitumor immune responses due to their robust self-renewal and proliferative potential. Emerging evidence indicates that the transcription factor MYB serves as a central regulator of T cell exhaustion dynamics^[108]^. Importantly, MYB-dependent CD62L^+^ Tpex cells constitute the primary population undergoing clonal expansion in response to PD-1 checkpoint blockade. This discovery reveals a novel therapeutic strategy to overcome immunotherapy resistance by modulating MYB activity to preserve the Tpex cell pool, thereby enhancing the durability and efficacy of immune-based treatments.

#### T cell mitochondrial dysfunction

T cell activation is strictly regulated by cellular metabolism^[109]^. Mitochondrial dysfunction represents a central driver of T cell exhaustion. Chronic antigen stimulation diminishes mitochondrial quality and impairs oxidative phosphorylation (OXPHOS) in T cells, leading to insufficient ATP production and accelerated functional exhaustion and apoptosis^[110]^. Tumor cells exacerbate this process by extracting healthy mitochondria from T cells via tunneling nanotubes while transferring mutated/dysfunctional mitochondria to them collectively promoting metabolic collapse and exhaustion^[111]^. Genetic absence of Slc25a3 in CD8^+^ T cells induces profound metabolic reprogramming, manifested by a shift from mitochondrial respiration to aerobic glycolysis (Warburg effect^[112]^), resulting in inefficient energy utilization and accelerated differentiation of Tpex to Tex-term cells^[113]^. Therapeutic strategies targeting mitochondrial restoration (e.g., engineered PGC-1α reprogramming^[114]^, currently in early-phase clinical trials) may effectively reverse exhaustion and enhance effector function, offering novel metabolic interventions to optimize cancer immunotherapy.

Chronic antigen stimulation reduces activity of acyl-CoA synthetase short-chain family member 2 in T cells, decreasing acetyl-CoA production and subsequent histone acetylation. This epigenetic remodeling impairs effector gene expression and self-renewal capacity in Tpex, ultimately promoting terminal exhaustion and ICI resistance^[115]^. This discovery suggests that targeting the metabolic–epigenetic axis could overcome ICI resistance.

Amino acid metabolism critically regulates T cell function at multiple levels^[116,117]^. Indoleamine 2,3-dioxygenase 1 (IDO1), the rate-limiting enzyme in tryptophan catabolism, is overexpressed by tumor cells and immunosuppressive cells in response to inflammatory signals. IDO1 activation depletes tryptophan to directly induce T cell exhaustion while simultaneously promoting expansion of Tregs and MDSCs, thereby establishing an immunosuppressive microenvironment^[118,119]^. Although IDO1 inhibitors combined with ICIs showed promising results in phase I/II trials^[120,121]^, a phase III melanoma study demonstrated no significant progression-free or overall survival benefit compared to pembrolizumab monotherapy^[122]^.

In CRC, metabolic alterations in the TME lead to ammonia accumulation. Bell *et al.* demonstrated that elevated ammonia levels drive metabolic reprogramming and exhaustion in T cells^[123]^. Their spontaneous CRC metastasis model revealed that enhancing ammonia detoxification can reactivate T cells and synergize with anti-PD-L1 therapy, providing a novel strategy to overcome immunotherapy resistance.

#### Irreversible epigenetic reprogramming of exhausted T cells

Epigenetic reprogramming represents a fundamental determinant of T cell exhaustion, primarily mediated through chromatin architecture alterations and dysregulated DNA/histone methylation^[124,125]^. The transcription factor TOX activates exhaustion-related enhancers while suppressing effector genes by recruiting chromatin remodeling complexes (e.g., INO80 and BAF)^[126]^. Loss of TOX can partially restore Tpex effector function, but its role in Tex-term cells may be constrained by irreversible DNA methylation. Exhausted T cells exhibit de novo methylation at key loci (e.g., PD-1 promoter, TCF7, IFN-γ, and Myc)^[127,128]^, which locks in the exhausted state.

The DNA methyltransferase (DNMT) inhibitor decitabine demonstrates synergistic efficacy with PD-1 inhibitors (e.g., camrelizumab) in advanced NSCLC by reversing PD-1 promoter hypermethylation^[129,130]^. Concurrently, histone acetylation dynamics mediated by NFAT and BATF transcription factors further sculpt the exhaustion epigenome^[131]^. Metabolic perturbations in exhausted T cells exacerbate these modifications through altered acetyl-CoA and α-ketoglutarate levels, establishing a self-reinforcing cycle of epigenetic dysregulation^[132]^.

**Figure 4:**
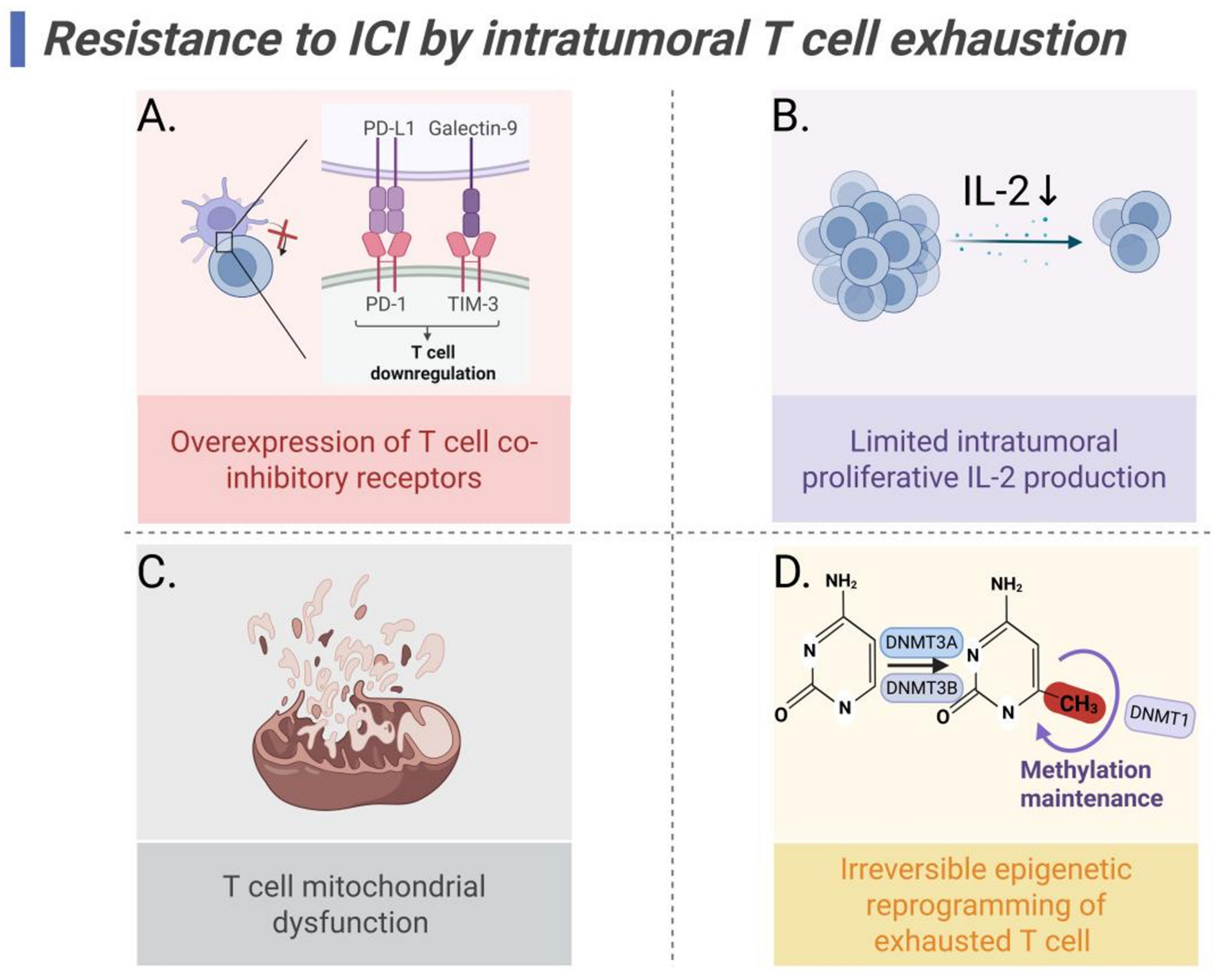
Mechanisms of ICI resistance mediated by T cell exhaustion. (A) Overexpression of T cell co-inhibitory receptors induces TCR signal blocking; (B) Reduced IL-2 production limits T cell proliferation; (C) T cell mitochondrial dysfunction; (D) DNA methylation causes exhausted T cell irreversible epigenetic reprogramming. Created in BioRender. Wang, L. (2025) https://BioRender.com/a1sc7r0. ICI: Immune checkpoint inhibitor; TCR: T cell receptor; IL-2: interleukin-2; PD-L1: programmed death-ligand 1; PD-1: programmed cell death protein 1; TIM-3: T cell immunoglobulin and mucin domain 3.

Importantly, these epigenetic features persist even after antigen clearance. Open chromatin regions keep “epigenetic scars”, which prevent full T cell recovery^[133,134]^. This explains why PD-1/PD-L1 blockade often produces only transient benefits. While epigenetic therapies show promise, challenges remain: DNMT inhibitors may induce genomic instability through off-target effects, and the field lacks exhaustion-specific epigenetic modulators. Future research needs to combine single-cell epigenomics and multi-omics analysis to decipher the specific epigenetic signatures of exhausted subpopulations, develop reversible regulatory tools, and achieve “on-demand activation” of T cell function [Figure 4].

In summary, T cell dysfunction emerges as a central mechanism of ICI resistance. Understanding these diverse, interlinked processes is critical for the development of next-generation immunotherapies. These factors collectively shape the immunosuppressive landscape of the TME and underscore the urgent need for combinatorial strategies to restore robust and durable T cell antitumor responses [Table 1].

**Table 1:** Mechanisms of T cell dysfunction-mediated ICI resistance and corresponding therapeutic strategies

**ICI resistance mechanism**			**Target/therapeutic strategy**	**Species**	**Cancer type**	**Stage of research**
T cell dysfunction	Excluded T cell tumor infiltration	Chemokine recruitment dysregulation	Anti-CSFR1 + anti-PD-1^[34]^	Mice	Melanoma	Preclinical research
		Intratumoral vascular structural abnormalities	LMWH + anti-PD-1^[37]^	Mice	CRC	Preclinical research
		Carcinogenic signaling pathways constrain T cell infiltration	Anti-CDK4/6 + anti-PD-1^[19]^	Mice	Melanoma	Preclinical research
		ECM fibrosis confines T cell penetration	Anti-Nox4 + anti-PD-1^[45]^	Mice	CRC/LC/BC	Preclinical research
		Intratumoral metabolic stress suppresses T cell infiltration	Anti-PI3K-γ + anti-PD-1^[135]^	Human	NSCLC/BC/melanoma	Phase I/Ib trial (NCT02637531)
	Defective TAA recognition	Low immunogenicity of TAAs	Anti-PD-1 + peptide neoantigen vaccines^[136]^	Human	Melanoma	Phase I trial (NCT01970358)
		Defective MHC-I antigen presentation	/	/	/	/
		Intratumoral dysregulated interferon signaling pathway	IFNα + anti-PD-1^[87]^	Human/mice	HCC	Preclinical research
	Intratumoral T cell exhaustion	Overexpression of T cell co-inhibitory receptors	Anti-LAG3 + anti-PD-1^[105]^	Human	Melanoma	Phase II trial (NCT03743766)
		Limited intratumoral proliferative IL-2 production	IL-2 + anti-PD-1^[16]^	Human	RCC/NSCLC/melanoma	Phase I/Ib trial (NCT02983045)
		T cell mitochondrial dysfunction	Anti-IDO1 + anti-PD-1^[137]^	Human	NSCLC/melanoma/RCC	Phase I/II trial (NCT02178722)
		Irreversible epigenetic reprogramming of exhausted T cells	Anti-DNMT + anti-PD-1^[129,130]^	Human	NSCLC	Phase I/II trial (NCT01799083)

ICI: Immune checkpoint inhibitor; PD-1: programmed cell death protein-1; LWMH: low molecular weight heparin; CRC: colorectal cancer; ECM: extracellular matrix; LC: lung cancer; BC: breast cancer; PI3K: phosphatidylinositol 3-kinase; NSCLC: non-small cell lung cancer; TAA: tumor-associated antigen; HCC: hepatocellular carcinoma; LAG3: lymphocyte activation gene 3; IL-2: interleukin-2; RCC: renal cell carcinoma; IDO1: indoleamine 2,3-dioxygenase 1; DNMT: DNA methyltransferase.

## MICROBIOTA-DRIVEN ICI RESISTANCE

While T cell dysfunction represents a central mechanism of ICI resistance within the TME, accumulating evidence highlights that resistance is not solely determined locally. Systemic host factors, particularly the gut microbiota, critically shape immune tone and therapeutic responsiveness. The gut microbiome engages in dynamic crosstalk with systemic physiology through microbial metabolites [short-chain fatty acids (SCFAs), bile acids, tryptophan derivatives] that circulate systemically or transmit neural signals to distant organs^[138,139]^. The gut ecosystem comprises bacteria, archaea, fungi, and viruses, typically dominated (> 90%) by Firmicutes (e.g., *Lactobacillus*, *Clostridium*) and Bacteroidetes (e.g., *Bacteroides*, *Prevotella*), with minor populations including Actinobacteria (e.g., *Bifidobacterium*) and Proteobacteria (e.g., *Escherichia*)^[140]^. In oncology, gut microbiota not only participate in drug metabolism^[141]^ but also modulate therapeutic responses by immunomodulation. High diversity of gut microbiota correlates with improved ICI responses, whereas microbial imbalance is associated with therapeutic resistance^[142,143]^. As a pivotal regulator of the TME and treatment response, the gut microbiota represents a promising target for overcoming treatment resistance^[144-146]^.

The gut microbiota systemically shapes the immune landscape of TDLNs, key hubs for antigen presentation and T cell priming. Circulating metabolites such as SCFAs and inosine reach TDLNs, where SCFAs, particularly butyrate and propionate, enhance DC antigen presentation and anti-inflammatory responses by suppressing lipopolysaccharide (LPS)-induced IL-6 and IL-12p40, while inosine serves as an alternative carbon source under glucose-limited conditions, supporting T cell proliferation and differentiation and increasing sensitivity to ICIs^[147,148]^. Conversely, dysbiosis-associated metabolites such as secondary bile acids impair TDLN immunogenicity by inducing tolerogenic DCs and expanding Tregs^[149]^. Thus, systemic microbial metabolites calibrate TDLN function and determine the quality of antitumor immunity, ultimately shaping ICI responsiveness.

The gut microbiota bidirectionally modulates ICI responses through multifaceted mechanisms. Microbial metabolites such as inosine enhance tumor immunogenicity by upregulating IFN-γ transcription in tumor cells, thereby improving antigen-presenting capacity and promoting T cell infiltration and activation^[143,150]^. SCFAs (e.g., butyrate, valerate) produced by colonic anaerobes can directly enhance the antitumor cytotoxicity of CD8^+^ T cells^[151]^. Molecular mimicry between microbial antigens and tumor antigens induces DC-mediated cross-reactivity of T cells, augmenting antitumor immunity^[152,153]^. The microbiota additionally modulates immune-related adverse events (irAEs) through immunoregulatory mechanisms; for instance, *Bifidobacterium intestinalis* promotes Treg expansion, while *Lactobacillus reuteri* may reduce type 3 innate lymphoid cells (ILC3s) populations to decrease excessive immune responses^[154,155]^. Furthermore, specific microbial taxa have been shown to enhance PD-1 blockade efficacy through PD-L2 downregulation^[156]^.

Conversely, microbiota-driven resistance mechanisms involve dysbiosis-induced immunosuppressive microenvironments. Antibiotic-mediated proliferation of multidrug-resistant bacteria activates inflammasomes, which exacerbate Treg infiltration while suppressing Teff cell function^[157]^. An altered ratio of *Bacteroidota* to *Firmicutes* has been associated with reduced PD-L1 expression and inferior treatment outcomes^[158]^. Therapeutic microbiota modulation through fecal microbiota transplantation (FMT) from ICI responders to refractory patients can increase the abundance of beneficial bacteria (e.g., *Ruminococcaceae*, *Bifidobacteriaceae*), reshaping the microbial composition, resulting in CD8^+^ T cell activation and reducing immunosuppressive populations. Clinical trials demonstrate that FMT combined with PD-1 inhibitors can effectively reverse primary resistance in advanced melanoma by remodeling the TME^[159-161]^, representing a promising strategy to overcome immunotherapy resistance.

## TARGETING TDLNS TO OVERCOME ICI RESISTANCE

Recent advances have redefined our understanding of TDLNs, not merely as passive sites of antigen presentation, but as active immunological hubs orchestrating T cell fate, functional plasticity, and responsiveness to ICIs^[6,10,162,163]^. Accumulating evidence suggests that TDLNs play dual roles in tumor immunity, as they can both initiate potent antitumor responses and contribute to therapeutic resistance^[9]^. Impaired DC migration, defective antigen presentation by APCs, and the priming of exhaustion-prone T cells within TDLNs can collectively lead to suboptimal immune activation and the emergence of ICI resistance. As such, immunological reprogramming of the LN microenvironment has emerged as a promising strategy to enhance the efficacy of cancer immunotherapy. A comprehensive understanding of the anatomy and immune function of LNs is essential for developing rational TDLN-targeted therapies.

### Anatomy and physiological functions of LNs

**Figure 5:**
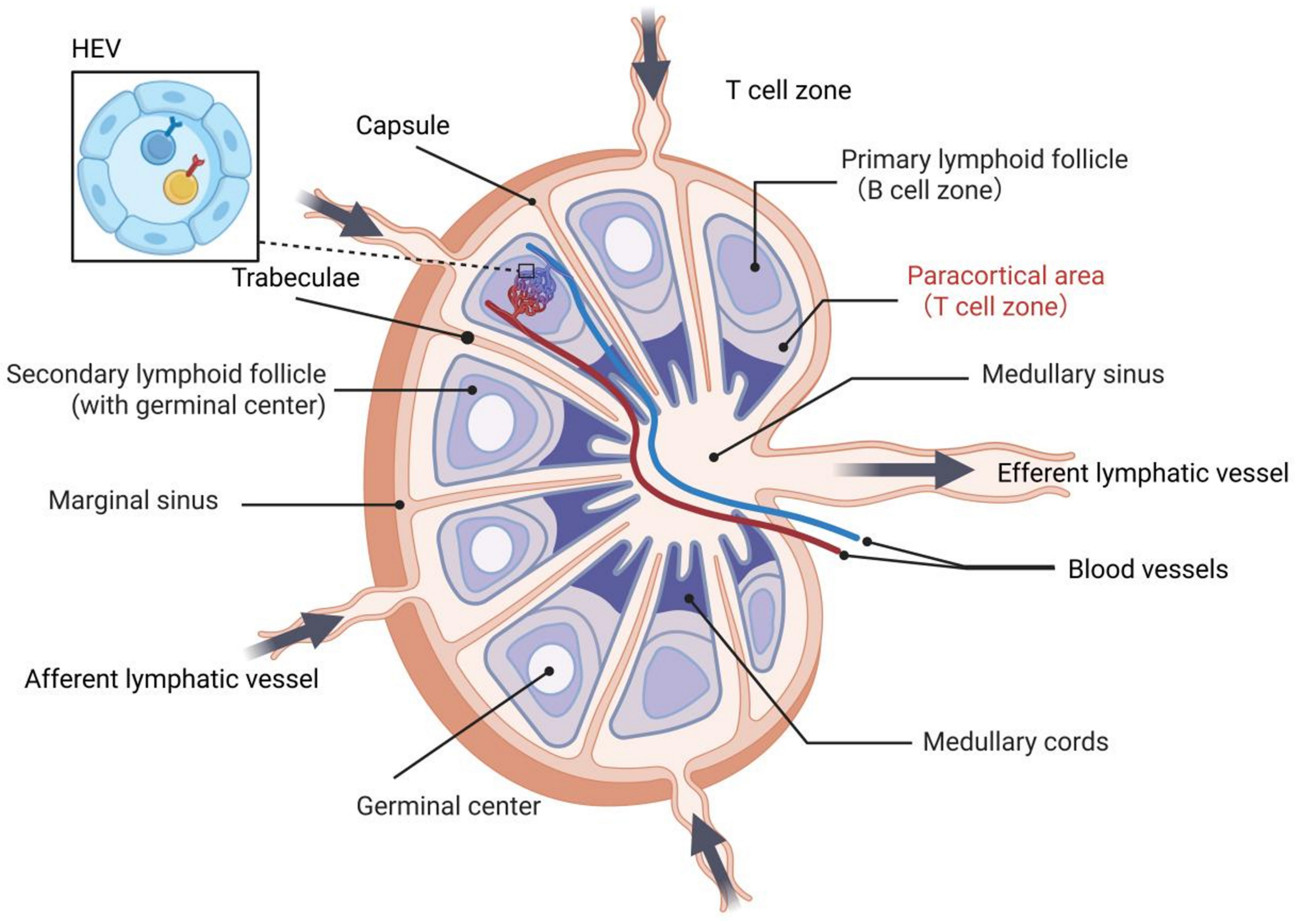
LN structure. The afferent lymphatic vessels transport antigens and APCs into the LN via the marginal sinus. The outer cortex contains the primary and secondary lymphoid follicles, including the germinal centers that support B cell activation and affinity maturation. The paracortical area (TCZ) is rich in T cells and HEVs, facilitating lymphocyte entry from the bloodstream. The medullary region, comprised of the medullary cords and sinuses, serves as a conduit for lymph and cellular egress through the efferent lymphatic vessel. Created in BioRender. Wang, L. (2025) https://BioRender.com/m0i16bb. LN: Lymph node; APCs: antigen-presenting cells; TCZ: T cell zone; HEVs: high endothelial venules.

LNs constitute essential SLOs that are strategically distributed throughout the body to form an integrated immunological surveillance network. These bean-shaped or ovoid structures exhibit complex, highly organized architecture divided into distinct functional compartments [Figure 5]. Each LN is encapsulated by a dense connective tissue^[164]^, from which trabeculae extend inward to form a reticular framework that supports vascular and neural distributions. The parenchyma consists of peripheral cortical and central medullary regions without distinct demarcation^[165]^. The superficial cortex (subcapsular region) represents the primary B cell zone (BCZ), while the deep cortex (paracortical region) between the superficial cortex and medulla serves as the predominant T cell zone (TCZ) containing high endothelial venules (HEVs) that mediate lymphocyte extravasation from circulation^[166]^. The medulla, located centrally, comprises lymphocyte-rich medullary cords and sinuses that facilitate lymph flow into efferent lymphatic vessels. Through precise spatial partitioning and intricate cell interactions, LNs achieve efficient filtration of lymph fluid^[167]^, antigen presentation and immune response^[168]^, and precise regulation of lymphocyte recirculation^[169]^.

#### LNs as training camps for antitumor T cells

Antigen-inexperienced naïve T cells that have completed thymic development migrate from circulation into the TCZ of LNs through HEVs guided by CCR7-dependent chemotaxis toward CCL19/CCL21 gradients^[170,171]^. This homing process involves sequential molecular interactions: initial tethering and rolling mediated by L-selectin (CD62L) binding to vascular addressins on HEVs, followed by chemokine-triggered (particularly CCL19/CCL21^[172]^) activation of lymphocyte function-associated antigen-1 (LFA-1, CD11a/CD18) that establishes firm adhesion through high-affinity binding to intercellular adhesion molecule-1 (ICAM-1) on endothelial cells^[173]^. These interactions culminate in transendothelial migration into the paracortical region^[169]^.

DCs capture antigens from peripheral tissues and, under the influence of chemokines such as CCL19/CCL21, enter the subcapsular sinus of the LNs via the afferent lymphatic vessels^[174,175]^. Both migrating and LN-resident DCs then use CCR7 to follow stromal cell-derived CCL19/CCL21 chemokine gradients, localizing in the TCZ. There, they process and present antigenic peptides via MHC-I/II molecules^[176]^. Naïve T cells systematically scan DC surfaces in a random walk pattern facilitated by the fibroblastic reticular cell (FRC) network and chemotactic signals^[177,178]^. Initial transient contacts with antigen-bearing DCs transition to stable immunological synapses when sufficient TCR clustering and sustained signaling occur, complemented by co-stimulatory interactions (e.g., CD28-B7 binding)^[179,180]^. This activation triggers clonal expansion and differentiation into Teff cells that either egress via efferent lymphatics to peripheral tissues or form LN Trm for sustained immune surveillance^[7]^. Beyond these canonical pathways, additional cellular interactions and signaling axes further shape T cell fate within TDLNs. M2-like macrophage-derived IL-10/TGF-β can impose suppression^[181]^, while stromal and lymphatic endothelial cells contribute to niche remodeling through the VEGF-C-VEGFR3 axis, which promotes lymphangiogenesis but may also facilitate immune evasion^[182]^. Moreover, co-stimulatory and co-inhibitory pathways, including ICOS-ICOSL and PD-1-PD-L1, operate within TDLNs to fine-tune the balance between effector differentiation and Tex cells.

#### Bidirectional Immunomodulatory Roles of TDLNs

While TDLNs orchestrate the priming and fate decisions of antitumor T cells, their dysfunction transforms them into key drivers of immune ICI resistance^[183]^. In particular, impaired CCR7/CCL21-mediated DC migration and defective antigen presentation lead to insufficient priming of naïve T cells^[164,184]^, providing a mechanistic basis for primary resistance in patients. Moreover, vascular and lymphatic abnormalities in TDLNs not only disturb local immune architecture but also restrict the egress of activated Teff cells into tumors^[185]^, thereby exacerbating “immune-excluded” phenotypes that blunt ICI efficacy. Beyond these structural and migratory defects, the progressive exhaustion of Tpex cells in TDLNs diminishes the pool of cells capable of responding to checkpoint blockade, while immunosuppressive cytokines and metabolic mediators further reinforce acquired resistance. These findings support a unified framework in which TDLN dysfunction drives ICI resistance across multiple stages of the antitumor response, underscoring the need for therapeutic interventions that restore their immunological competence.

### Important roles of TDLN in the CI Cycle

**Figure 6:**
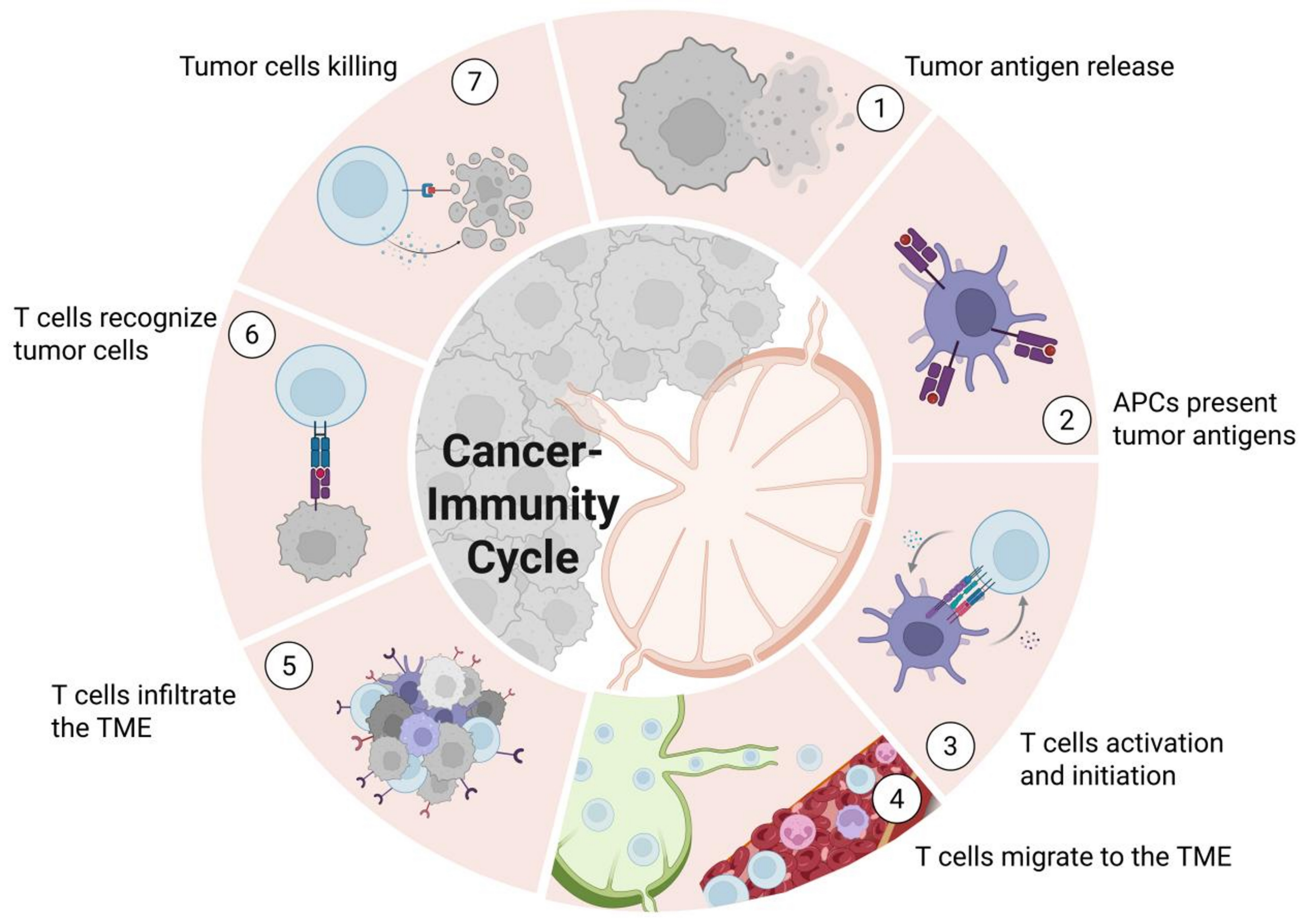
CI Cycle. ① Cancer cell death leads to the release of tumor antigens; ② DCs capture these antigens, process them, and present them on MHC molecules to T cells within TDLNs; ③ Naïve T cells recognize the antigens presented by DCs, become activated, and differentiate into Teff cells; ④ The activated T cells migrate from the TDLN through the bloodstream toward the site of the tumor; ⑤ The activated T cells extravasate across the endothelial barrier and infiltrate the TME; ⑥ The Teff cells recognize and bind to their specific antigens presented on the surface of cancer cells via MHC molecules; ⑦ The activated T cells release cytotoxic molecules that kill the target cancer cells, releasing more antigens and restarting the cycle. Created in BioRender. Wang, L. (2025) https://BioRender.com/aeg45x2. APCs: Antigen-presenting cells; CI Cycle: cancer-immunity cycle; DCs: dendritic cells; MHC: major histocompatibility complex; TDLNs: tumor-draining lymph nodes; Teff: effector T; TME: tumor microenvironment.

The CI Cycle, first conceptualized by Chen and Mellman in 2013^[186]^, describes the process by which the human immune system recognizes and eliminates tumor cells. This framework comprises seven sequential steps [Figure 6], including the release of tumor antigens, antigen presentation by APCs, activation and priming of T cells, trafficking of T cells to the TME, T cell infiltration, recognition of tumor cells, and subsequent tumor cell elimination. Each step involves distinct cell types and molecular mechanisms that collectively drive antitumor immunity.

As the starting and regulatory center of the CI Cycle, TDLNs are involved in many aspects of this process. During the antigen presentation phase (Step 2), DCs and other APCs, after capturing tumor antigens in the TME, migrate to TDLNs through lymphatics and present tumor antigens to T cells through MHC-I/II molecules^[187]^. The T cell activation phase (Step 3) occurs in the LNs, where specialized structures HEVs facilitate optimal T cell-APC interactions^[8]^, while co-stimulatory signals (e.g., CD80/CD86-CD28 engagement) drive the differentiation of naïve T cells into Teff cells. The LN cytokine milieu (e.g., IL-12, IFN-γ) further promotes clonal expansion and differentiation of tumor-specific cytotoxic T cells. Subsequently, during the T cell trafficking phase (Step 4), activated T cells exit through efferent lymphatics, enter systemic circulation, and home to tumor sites guided by chemokine gradients (e.g., CXCL9/10)^[188]^.

Beyond these canonical roles, LNs function as reservoirs for memory T cells. Successful antitumor immune responses establish long-term immune memory here, providing a basis for rapid response to subsequent tumor recurrence^[6]^. This immunological memory, maintained within LN niches, represents a fundamental mechanism for preventing disease relapse following immunotherapy.

### Targeting TDLNs to enhance T cell tumor infiltration

**Figure 7:**
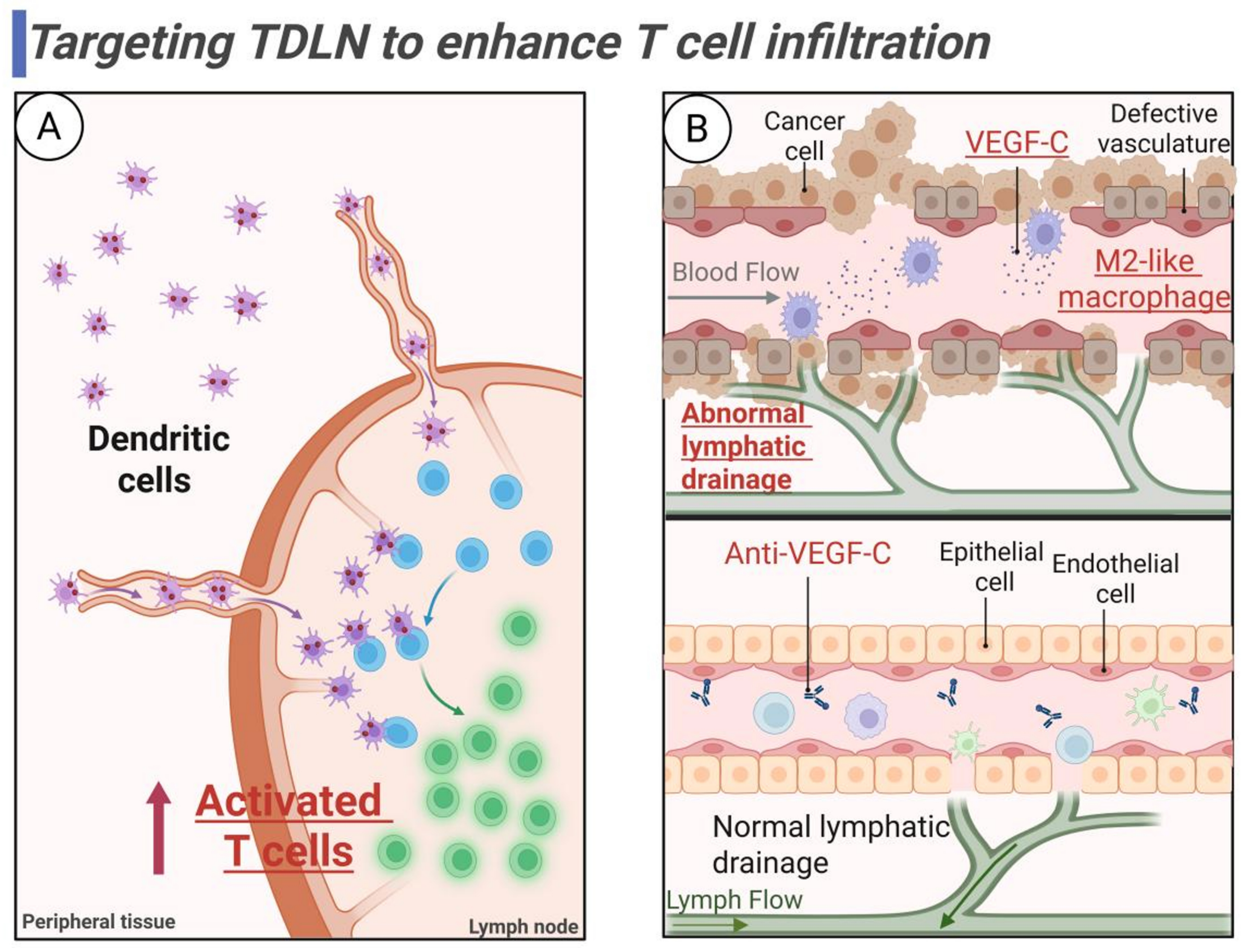
Targeting TDLN to enhance T cell infiltration. (A) Targeting DCs in TDLN to prime and activate T cells; (B) Targeting macrophages in TDLN to regulate vascular and lymphatic homeostasis for T cell migration. Created in BioRender. Wang, L. (2025) https://BioRender.com/cg0mqy5. TDLN: Tumor-draining lymph node; DCs: dendritic cells; VEGF-C: vascular endothelial growth factor-C.

As previously discussed, impaired T cell infiltration results from multifactorial interactions. This section focuses on TDLN-targeted strategies involving DCs and macrophages [Figure 7], particularly emphasizing that modulating chemokine pathways and remodeling vascular/lymphatic networks to enhance T cell tumor infiltration could potentially overcome ICI resistance.

#### Targeting DCs in TDLN to enhance antitumor T cell priming and activation

As the “sentinels” of the immune system, DCs dominate the immune response in TDLNs mainly through antigen presentation and chemokine regulation. These professional APCs drive antitumor immunity by activating naïve T cells and recruiting effector immune populations^[162,189]^. Precursor DCs (pre-DCs) in the LN medulla differentiate into mature cDC1/cDC2, forming a three-dimensional DC network structure that maintains immune surveillance functions^[190]^. Disruption of TDLN development, such as through FOXC2 deficiency, can compromise this DC migration network and lead to immune surveillance failure^[191]^. It is generally accepted that migratory DCs (e.g., cDC1) transport tumor antigens to LNs via specific chemokine receptors (e.g., CCL21/CCR7 signaling)^[164,184]^, which activate CD8^+^ T cells via cross-presentation, while the cDC2 subpopulation supports CD4^+^ T cell activation^[2,6,7]^. Interestingly, Dammeijer *et al.* found in mouse models that PD-L1^+^ cDC2s were a major target for anti-PD-L1 therapy in TDLNs, and that PD-L1^+^ cDC2s modulate CD8^+^ T cell function in a way that breaks with conventional knowledge, suggesting that targeting and modulating cDC2s can also enhance cancer immunotherapy^[163]^.

The immunomodulatory functions of DCs extend to their secretion of CCL8, which activates Src kinase signaling to potentiate CCR7-mediated T cell migration to the paracortical region^[170,171,192]^, thereby promoting immune cell aggregation and activation while suppressing tumor progression. Genetic modification strategies, such as AdRGD vector-mediated *CCR7* gene transduction, can enhance DC migratory capacity to TDLNs by upregulating CCR7 expression^[193]^. Furthermore, DC-derived chemokines, including CXCL9 and CXCL10, guide Teff cell trafficking to tumor sites through CXCR3 receptor binding, facilitating tumor infiltration^[194]^.

Recent advances in nanotherapeutic approaches have demonstrated the potential for precise DC targeting in TDLNs. Wang *et al.* demonstrated in melanoma mice that TLR7 agonists loaded into mesoporous polydopamine (MPDA) nanocarriers could directly target DCs in the TDLN, promote DC maturation and antigen-presenting ability, facilitate T cell activation and migration, and significantly enhance anti-PD-1 efficacy and reverse melanoma resistance in mice^[195]^. Targeting DCs within LNs not only enhances antigen presentation but also regulates chemokine axes to recruit Teff cells and promote their tumor infiltration, thereby augmenting antitumor immunity at multiple levels. This strategy provides a practical and promising approach to overcoming PD-1 resistance and improving the therapeutic efficacy of immune ICIs.

#### Targeting macrophages in TDLN to remodel vascular and lymphatic networks for T cell migration

LN macrophages serve as pivotal regulators of the TME, extending beyond their classical antigen-presenting and immune-activating functions to modulate T cell infiltration efficiency through vascular and lymphatic network remodeling. Targeted modulation of the function of these cells may disrupt the immunosuppressive barrier of TDLNs, thereby enhancing the immunotherapeutic response.

Macrophages in the TDLN are remarkably heterogeneous, with different subpopulations synergistically regulating lymphatic and vascular homeostasis through unique spatial distribution and functional properties. The major macrophages in the TDLN include subcapsular sinus macrophages and medullary sinus macrophages. Subcapsular sinus macrophages located in the subperitoneal sinus (SCS) of TDLNs are professional APCs that capture tumor antigens or tumor-derived extracellular vesicles (tEVs) in the lymph fluid via surface receptors (e.g., SIGN-R1, CD169). Similar to DCs, they secrete chemokines (e.g., CCL21) to recruit T cells and mediate CD8^+^ T cell activation through cross-presentation^[196-198]^. Furthermore, their surface expression of ICAM-1 and VCAM-1 enhances adhesion with B cells and promotes antigen-specific B cell activation^[199,200]^. These macrophages are also involved in the maintenance of LN structure, interacting with lymphatic endothelial cells (LECs) and FRCs to form a microenvironment for immune cell activity^[10]^.

Residing in the medullary sinuses, medullary sinus macrophages specialize in phagocytosing apoptotic tumor cells^[201]^ and presenting tumor antigens via MHC-I to maintain CD8^+^ T cell-mediated immune surveillance. Their production of IL-12 and IFN-γ polarizes Th1-type responses, amplifying antitumor immunity^[10,184]^.

Pathological remodeling of vascular and lymphatic structures in TDLNs constitutes a critical barrier limiting efficient T cell infiltration, where M2-like macrophage-derived VEGF-C drives aberrant lymphangiogenesis, resulting in the formation of leaky lymphatic networks that impair T cell migration into parenchymal regions^[185]^. The use of anti-VEGF-C (e.g., VGX-100) or low-dose anti-VEGFR-3 antibody (e.g., LY3022856) has been shown to reduce LEC hyperplasia, enhance T cell and DC migration efficiency, and restore structural integrity of lymphatic vessels and vascular normalization^[202-204]^. Not only that, targeting the PI3K-AKT-mTOR-HIF-1α signaling pathway also downregulated macrophage VEGF-C expression, while inhibiting the transformation of M1-like macrophages to LECs^[205]^.

Under hypoxic conditions characteristic of the TDLN microenvironment, inhibition of macrophage HIF-1α expression reduces secretion of VEGF-A and MMP-9, thereby reversing HEV constriction, improving vascular architecture, and augmenting T cell influx^[206]^. The development of nanoparticle (NP)-based delivery systems for precision targeting of HIF-1α inhibitors to LN-resident macrophages may represent a promising therapeutic strategy to enhance T cell tumor infiltration and overcome resistance to ICIs.

### Targeting TDLN with personalized vaccines to enhance TAA recognition by T cells

Recent advances in nanotechnology and tumor immunology have propelled personalized tumor antigen vaccines to become an important strategy to overcome immunotherapy resistance^[207]^. Encapsulating tumor-specific antigens or adjuvants into carriers such as liposomes or polymeric NPs can transform poorly immunogenic antigens into highly immunogenic signals. Through LN-targeted delivery, these engineered nanovaccines enable efficient activation of antitumor T cell immune responses. Xi *et al.*^[208]^ and Thomas *et al.*^[209]^ constructed an antigen-CpG self-assembled NPs, formed by electrostatic interaction, which significantly enhanced the antigen cross-presentation efficiency of melanoma mice LN-resident DCs and induced rapid CD8^+^ T cell activation. Peptide neoantigen vaccines targeting DCs by subcutaneous injection and activating T cells within LNs have shown significant antitumor effects in melanoma patients, with emerging evidence of synergistic efficacy when combined with PD-1 inhibitors^[136]^. Chu *et al.* employed a liposome-based delivery system to develop polymeric neoantigen nanovaccines that target APCs in TDLNs, demonstrating enhanced cross-presentation and subsequent activation of neoantigen-specific CD8^+^ T cell responses in the melanoma mice^[210]^. Moreover, the study by Chen *et al.* demonstrated that LN–targeted delivery of mRNA vaccines can enhance the immunogenicity of tumor antigens, resulting in a potent antitumor immune response in a murine melanoma model^[211]^.

These innovative approaches not only address the critical limitation of insufficient antigen presentation underlying ICI resistance but also exhibit synergistic potential when combined with PD-1/PD-L1 blockade therapy. Future optimization of nanovaccine delivery systems holds considerable promise for further improving T cell activation efficiency, overcoming the tumor immune escape, and providing more precise therapeutic solutions to alleviate ICI resistance.

### Expanding Tpex pools in TDLN to alleviate intratumoral T cell exhaustion

Stem-like Tpex cells are a unique subset of CD8^+^ T cells with distinct biological functions, which maintain the persistence of the antitumor T cell response through strong self-renewal, high proliferative potential, and multidirectional differentiation capacity^[212,213]^. They are mainly resident in the TCZ of the TDLNs and tertiary lymphatic structures (TLSs)^[213,214]^. Tpex highly expresses T cell factor-1 (TCF-1), PD-1, and CXCR5, underscoring their stem-like properties. Among them, TCF-1 (encoded by TCF7) is a master regulator of Tpex stemness and synergistically drives stem cell properties with Bcl-6^[69,215-217]^. The proportion of TCF-1^+^ T cells in tumors is strongly associated with clinical prognosis^[69,99,218]^. Multiple studies have demonstrated that an increased Tpex population often predicts improved survival following ICI therapy^[69,99,108,219,220]^. Under multi-layered regulation involving transcriptional networks, metabolic reprogramming, epigenetic modifications, and microenvironmental cues, Tpex (PD-1^+^, TCF-1^+^, CXCR5^+^) progressively differentiate into Tex-term (Tim3^+^, CD39^+^, TCF-1^-^), which exhibit diminished proliferative capacity and cytokine secretion.

Interleukin-7 (IL-7) sustains Tpex stemness by upregulating TCF-1 via signal transducer and activator of transcription 5 (STAT5) activation. In preclinical studies, the combined administration of recombinant human interleukin (rhIL)-7-hyFc (efineptakin alfa; NT-I7), hIL-2/TCB2 complex (SLC-3010), and PD-1 inhibitor robustly promoted the expansion of Tpex cells in TDLNs of a melanoma model and facilitated their differentiation into a highly cytotoxic effector subpopulation, achieving complete tumor regression^[221]^. Similarly, IL-15 promotes Tpex proliferation and inhibits apoptosis by JAK-STAT signaling. In a chronic infection model and RCC patients, exogenous IL-15 significantly increased the proportion of Tpex in TDLNs while enhancing their ability to differentiate into effector subpopulations^[222]^. Li *et al.* demonstrated in a mouse tumor model that the demethylating drug desmethylated cytarabine remodeled Tpex cells, driving differentiation into a more toxic-killing Teff cell subpopulation, and when combined with the single-use anti-PD-1 treatment, significantly increased therapeutic efficacy^[223]^. Terminally exhausted T cells highly express 4-1BB, and its agonist markedly enhances effector functions. Preclinical models revealed that combining 4-1BB agonism with PD-1 blockade reprograms terminally exhausted subsets, upregulates cytotoxic molecule expression, delays exhaustion progression, and suppresses tumor growth^[224,225]^.

As a key regulator of antitumor immune response, the stemness maintenance and differentiation fate of Tpex directly affect the long-term effects of immunotherapy. The combination of “source intervention” (targeting Tpex) and “terminal activation” (PD-1 blockade) not only overcomes the resistance bottleneck of existing ICI therapy, but also establishes a long-lasting immune memory, providing a novel therapeutic paradigm to improve outcomes in solid tumors. Future development of precise modulation strategies targeting Tpex-specific markers (e.g., TCF-1) will further enhance the clinical translational potential of combination therapy.

### Potential applications of TDLN–derived Tpex for adoptive T cell therapy

Advancement of adoptive cell therapy (ACT) has advanced significantly, and TIL therapy - the first U.S. FDA-approved ACT for solid tumors^[226]^ - has demonstrated promising efficacy in various malignancies^[227-230]^, particularly in melanoma^[231]^ and advanced cervical cancer^[232]^. Owing to their natural tumor specificity, TILs have become a central component of personalized immunotherapy. However, conventional TIL therapy predominantly relies on Teff cells isolated from tumor tissue, which are often enriched for Texh-term cells. These cells exhibit limited functional capacity and poor proliferative potential *ex vivo*, thereby seriously constraining the efficacy and persistence of immunotherapy^[233]^.

As previously discussed, TDLNs are enriched with TCF-1^+^ Tpex, which possess high proliferative potential, functional plasticity, and long-lived memory characteristics. Importantly, Tpex cells represent a key responsive population to PD-1 blockade^[69,99]^. This provides a new cell source option for TIL therapy. To overcome the limitations of conventional TIL therapy, TDLNs are considered a valuable supplementary source for the acquisition of TILs^[234]^. Specifically, selective enrichment and expansion of Tpex cells or early-activated CD8^+^ T cells from TDLNs may yield T cell products with superior proliferative capacity and functional persistence compared to conventional TILs. As LNs serve as central hubs for T cell priming and fate determination, Tpex cells derived from these sites are less antigen-exhausted and retain a more favorable metabolic and epigenetic landscape. We term this approach “TDLN-derived Tpex”, characterized by high Tpex content, enhanced expansion potential, and a greater capacity for effector differentiation. There have already been reports that CRC patients receiving ACT of sentinel lymph node (SLN)-derived lymphocytes achieved favorable therapeutic outcomes in Phase I/II clinical trials^[235]^. Moreover, a Phase I/II clinical trial of TDLN-derived lymphocytes ACT in patients with advanced HER2-negative BC is currently ongoing [NCT05981001], further highlighting the translational potential of this strategy. When integrated with gene-engineering strategies to enhance tumor homing and cytotoxicity, this approach may delay exhaustion and improve therapeutic durability.

Looking ahead, the precise enrichment and quality control of TDLN-derived Tpex can be achieved through the use of Tpex-specific surface markers (e.g., TCF-1^+^, CXCR5^+^, PD-1^+^^[69,217]^), optimization of the cytokine milieu (e.g., IL-2 and IL-15 to promote proliferation and chemokine-directed trafficking^[222,236]^), and TCR-seq analysis to ensure antigen specificity and clonotypic diversity. This strategy provides a novel therapeutic avenue for patients with ICI resistance, particularly those with immunologically “cold” tumors. The combination of TDLN-derived Tpex infusion and ICI therapy may potentiate synergistic antitumor immunity and expand the clinical applicability of TIL therapy to previously unresponsive tumor types.

Despite the promising therapeutic potential of targeting TDLNs and harnessing TDLN-derived Tpex, several limitations of the current strategies should be acknowledged. First, most proposed approaches are still supported primarily by preclinical evidence, with limited clinical validation. Although early-phase clinical trials of lymphocytes derived from TDLNs (e.g., SLNs) have shown encouraging results, the efficacy and safety of combining TDLN-derived Tpex with ICIs in solid tumors remain underexplored. Second, there exists considerable heterogeneity in TDLN structure and function across cancer types (e.g., melanoma *vs.* glioblastoma), which may critically influence therapeutic responses, thereby requiring optimization specific to each cancer type. Third, practical challenges of TDLN targeting must be addressed, including structural damage caused by LN metastasis and difficulties in accessing TDLNs in advanced disease. Innovative solutions, such as nanocarrier-mediated drug delivery or minimally invasive sampling approaches, may help overcome these barriers and expand clinical applicability.

**Figure 8:**
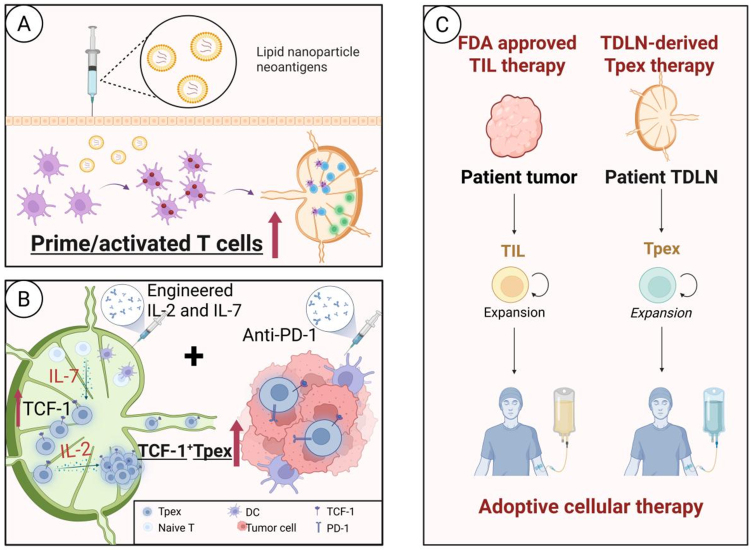
Targeting TDLN to overcome therapy resistance. (A) Personalized vaccines targeting TDLN enhance the antigen recognition ability of T cells to prime and activate T cells; (B) IL-7 maintains Tpex stemness via STAT5/PI3K-AKT signaling, whereas engineered IL-2 drives Tpex expansion and activation. The engineered IL-2 and IL-7 promote the proliferation of Tpex cells to alleviate T cell exhaustion, and their combination with anti-PD-1 can enhance the efficacy of PD-1 blockade; (C) Potential application of TDLN–derived Tpex cells for adoptive cellular therapy. Created in BioRender. Wang, L. (2025) https://BioRender.com/owk0csp. TDLN: Tumor-draining lymph node; IL-7: interleukin-7; Tpex: progenitor exhausted T cells; STAT5: signal transducer and activator of transcription 5; PI3K: phosphatidylinositol 3-kinase; AKT: protein kinase B; IL-2: interleukin-2; PD-1: programmed cell death protein 1; TCF-1: T cell factor-1; TIL: tumor-infiltrating lymphocyte.

In summary, we highlight the central role of LNs in shaping antitumor immunity and overcoming resistance to checkpoint therapy. By targeting DCs and macrophages in TDLNs, optimizing antigen presentation, and harnessing the Tpex or early-activated CD8^+^ T cells for ACT, these strategies collectively restore Teff cell priming, trafficking, and persistence [Figures 7 and 8]. We hope that these approaches can overcome the immunosuppressive constraints associated with checkpoint resistance, thereby restoring effective antitumor immune responses.

## CONCLUSION

Overcoming resistance to ICI therapy has emerged as a central challenge in cancer immunotherapy. This review highlights the pivotal role of T cells in antitumor immunity, systematically dissecting the complex multi-dimensional regulatory networks underlying resistance mechanisms across various stages of the T cell response, including infiltration, recognition, activation, and exhaustion. We emphasize the synergistic contributions of metabolic reprogramming, epigenetic regulation, and dysregulated interferon signaling within the TME to T cell dysfunction. Furthermore, we propose that LNs, as central hubs in antitumor immunity, warrant greater attention for their role in determining T cell fate. Targeted modulation of key immune cells (e.g., DCs, macrophages, and Tpex) in TDLNs, through metabolic or epigenetic interventions and spatial reorganization, may enhance T cell priming and functional persistence, thereby improving ICI efficacy.

“TDLN-derived Tpex” strategy leverages Tpex cells from TDLNs for *ex vivo* expansion and functional enhancement, followed by autologous reinfusion alongside ICI therapy, potentially providing a new avenue for optimizing and broadening the clinical utility of TIL-based immunotherapy. Future research should integrate single-cell multi-omics and spatial multi-omics to explore the functional spectrum of immune cell subsets in LNs, facilitating a paradigm shift from “tumor-targeted” to “immune-hub-targeted” therapeutic strategies.

## DECLARATIONS

### Copyright

© The Author(s) 2025.
